# Fasting blood glucose, blood pressure and body mass index among combined oral contraceptive users in Chencha town Southern Ethiopia, 2019

**DOI:** 10.3389/fgwh.2023.992750

**Published:** 2023-04-28

**Authors:** Zelalem Kofole, Diresbachew Haile, Yerukneh Solomon

**Affiliations:** ^1^ Department of Biomedical Sciences, School of Medicine, College of Health Science, Arba Minch University, Arba Minch, Ethiopia; ^2^ Department of Physiology, School of Medicine, College of Health Science, Addis Ababa University, Addis Ababa, Ethiopian; ^3^ Department of Biomedical Sciences, College of Medicine and Health Sciences, Debre Berhan University, Debre Berhan, Ethiopia

**Keywords:** contraceptives, pills, BMI, family planning, chencha

## Abstract

**Background:**

The use of contraceptives has become prevalent among women in Ethiopia. Oral contraceptive use has been suggested to trigger changes in glucose metabolism, energy expenditure, blood pressure, and body weight, among various populations and ethnic groups.

**Objective:**

To elucidate the pattern of fasting blood glucose, blood pressure, and body mass index among combined oral contraceptive pills users compared to controls.

**Methods:**

An institution-based cross-sectional study design was employed. A total of 110 healthy women using combined oral contraceptive pills were recruited as cases. Another 110 healthy age and sex-matched women not using any hormonal contraceptives were recruited as controls. A study was conducted between October 2018 and January 2019. Data obtained was entered and analyzed using IBM SPSS version 23 software packages. One-way ANOVA was used to identify the variation of variables in relation to the duration of use of the drug. The *P*-value of < 0.05 at the 95% confidence level was statistically significant.

**Results:**

Fasting blood glucose level among oral contraceptive users (88.55 ± 7.89 mg/dl) was higher than fasting blood glucose level among oral contraceptive non-users (86.00 ± 9.85 mg/dl) with a *p*-value of 0.025. The mean arterial pressure of oral contraceptive users (88.2 ± 8.48 mmHg) was relatively higher than their counterparts (86.0 ± 6.74 mmHg), with a *p*-value of 0.04. Comparatively the body weight and body mass index among oral contraceptive users were 2.5% and 3.9% higher than non-users with a *p*-value of 0.03 and 0.003, respectively(5). Utilization of oral contraceptive pills for prolonged period of time seemed to be a significant predictive factor for higher mean arterial pressure and body mass index with *p* < 0.001and *p* < 0.05 respectively.

**Conclusions:**

Use of combined oral contraceptives was associated with higher fasting blood glucose (+2.9%), mean arterial pressure (+2.5%), and body mass index (3.9%) compared to controls.

## Introduction

Oral contraceptive pills (OCPs) are one of the most common worldwide used family planning methods for preventing pregnancy and it was introduced in the early 1960s (1). Introduction of modern contraceptives has contributed significantly to avert maternal mortality by promoting the wellbeing of mothers and children (2). Modern contraceptives whether hormonal or non-hormonal, are medical procedures or products that interfere with reproduction following sexual intercourse (3). Among different methods of contraception, OCP use is common because of various factors such as reversibility, lower side effects and also its role in reducing risk of dysmenorrhea and ovarian cancers (4). Approximately one hundred million women use combined hormonal contraceptives worldwide, which are most used in the Western world (5). This could make hormonal contraceptives one of the prescription drugs most widely used in the world (5). The main contraceptive effect of OCP is inhibition of the hypothalamus-pituitary-ovarian axis *via* negative feedback mechanism; which is designed to suppress the secretion of pituitary gonadotropins, which, in turn, prevents follicular maturation, ovulation, and subsequently prevents pregnancy (6). Despite their benefits, hormonal contraceptive use may increase the risk of different disorders such as weight gain, hypertension, hyperglycemia, and increased BMI (7–9). Study report in Park et al. investigated that utilization of OCP for more than 24 months caused approximately two folds increase in the risk of developing hypertension (7). According to Liu et al., highest doses of OCP compared to lower doses caused approximately 50% higher risk of developing hypertension (10). Furthermore, a study from China also reported that OCP users had a nearly 40% higher risk of developing hypertension compared to OCP non users (11). In addition, clinical and experimental studies have reported that hormonal contraceptives deteriorate carbohydrate metabolism and insulin secretion of users (12, 13). Gonzale et al. investigated the influence of estradiol on insulin receptors, in which it was reported that estradiol cause the carbohydrate mechanism to deteriorate and decrease insulin sensitivity (8). These changes detected on carbohydrate mechanism are dependent on ethyl estradiol doses and androgenic effect of progestin (13). Besides the negative effects on blood pressure and carbohydrate metabolism, use of OCP has also been couple to weight gain and obesity (9). According to a study from Kenya, women who use OCP were 1.24 times more likely to be obese compared to OCP non-users (14). Similarly a study from Myanmar reported that the chance of being obese was 1.43 times more likely among OCP users as compared to their counterparts (15). Available data on effects of OCP use on blood pressure, carbohydrate metabolism, and body weight is mostly based on women from developed countries and less frequently from Africans. Specifically, no previous studies have investigated the association between OCPs use and fasting blood glucose (FBG), blood pressure, and body mass index (BMI) among users in Ethiopia. Therefore, we aimed to assess the association between OCPs use and fasting blood glucose, blood pressure, and body mass index.

## Methods

### Study area and period

The study was conducted at family planning services provider health centers in Chencha town, from October 2018 to January 2019.

### Study design and subjects

An institution-based cross-sectional study design was employed on 220 subjects in two groups. 110 healthy women who had been using OCPs were grouped as cases and 110 healthy age and sex matched controls that were not using any hormonal contraceptive.

### Eligibility criteria

Aged between 18 and 45 years; clinically stable and OCP users for more than 3 months included in the study as cases and healthy women who have the same inclusion criteria as users, but not using pills were included as controls. On the other hand, chronic alcohol and/or tobacco use; pregnancy; breastfeeding; women who were using other hormonal contraceptives; women who did not had full information on patient chart about diagnosis of chronic co-morbidities such us (thromboembolic disorders, cardiovascular disorders like hypertension, cerebrovascular accident like stroke, chronic renal disease, chronic liver disease, diabetes mellitus) before starting OCP were excluded from the study.

### Sample size determination and sampling

The two-sided population proportion formula was utilized for the determination of sample size. Taking prevalence of hypertension among OCP users was 35% and among controls was 18% (7); 95% confidence interval with a 5% margin of error. By adding a 10% non-response rate the final sample size was 220. A simple random sampling technique was applied to select 110 cases. Similar numbers of controls (110) were selected by convenient sampling from a similar population.

### Data collection procedure

A structured questionnaire that is a modified form of that of the World Health Organization (WHO) was used for the purpose (16). A pretested questionnaire pertinent to the study objectives was developed and used. All women were screened for eligibility based on a medical interview, and those who volunteered to participate in the study were recruited. Study participants answered questions in the questionnaire that were relevant to their socio-demographics. Afterward, systolic blood pressure (SBP) and diastolic blood pressure (DBP) were measured and mean arterial pressure (MAP) was calculated. The height and weight of each participant were measured to compute the BMI. A capillary blood sample was collected from each participant following overnight fasting for estimation of FBG.

### Anthropometric measurements

The height and weight of the study participants were measured. The height was measured without shoes using a meter rule and approximated to the nearest 1 cm. Weight was measured using the weighing scale nearest to 1 kg, with light clothing, and without phones or any encumbrance that could alter their appropriate weight. BMI was calculated using the following formula: weight/squared height in kg/m^2^.

### Blood pressure measurement

The resting blood pressure of study participants was measured by using a recently calibrated digital sphygmomanometer. Measurements were done after 15 min of rest in a sitting position three times, giving 3 to 5 min of rest in between the measurements in the left arm. According to WHO guidelines, the mean of the 2nd and 3rd readings was taken for data analysis. The SBP and DBP readings were taken from a digital sphygmomanometer. The MAP was calculated as DBP + 1/3 (SBP- DBP) from SBP and DBP (17).

### Data processing and analysis

SPSS software version 23 was used for data management and statistical analysis. Standard statistical methods were used to determine the mean, standard deviation, and range. An independent samples *t*-test was used to compare the results of the FBG level, MAP, and BMI of the cases with the control group. A one-way ANOVA was used to identify the variation of variables in relation to the duration of use of the drug. The *P*-value of < 0.05 at the 95% confidence level was statistically significant.

### Ethical considerations

Our study has been approved by the departmental research and ethical committee (DRC) of the college of health sciences at Tikur Anbessa Hospital, Addis Ababa University with protocol number Anat/Phy/256/2018, September 13/2018. All participants were informed of the objective of the study and provided written approval of it.

### Operational definitions

**Blood pressure:** The force exerted against blood vessel walls can be categorized as normal pressure below 120/80 mmHg, elevated systolic blood pressure between 120 and 129, and diastolic less than 80 mmHg, and hypertensive above 130/80 mmHg (17).

**Body Mass Index (BMI):** The ratio of body mass in kilograms to body height squared meter, expressed in kg/m^2^ units (18).

**Oral Contraceptive (OCP):** Often referred to as the birth control pill or colloquially as “the pill”, is a type of birth control that is designed to be taken orally by women. It includes a combination of estrogen and progesterone and alters the menstrual cycle to inhibit ovulation and prevent pregnancy (19).

**Fasting Blood Glucose (FBG):** Glucose level is the result of blood samples taken after the client fasts for at least eight hours or overnight, with a normal level less than 110 mg/dl, impaired fasting glucose (IFG) between 110 and 125 mg/dl and DM over 126 mg/dl (20).

## Results

### Socio demographic characteristics the study participants

A total of 220 study participants (110 OCP users and 110 controls) were included in the study. Most participants (99% of cases and 80% of controls) were married. Sixty-four (58.2%) of OCP users and 52 (47.3%) of the control groups had monthly incomes between 500 and 1,500 Ethiopian Birr. The mean age (years) of the two groups was 29.6 ± 3.56 (OCP users) and 28.3 ± 3.86 (control). The majority of the study participants; 70 (63.6%) of OCP users and 82 (74.5%) of the controls, were aged 20–30 years. The main religion was Orthodox Christian, with 62 (56.4%) OCP users and 57 (51.8%) controls. A significant number of subjects (23.6% of OCP users and 60% of controls) attended modern education, ranging from primary up to tertiary level. About 55 (50%) of OCP users and 38 (34.4%) of controls were homemakers (as shown in Table 1).

**Table 1:** Socio demographic characteristics of the study Participants.

Variables	Parameters	Cases (*n* = 110)	%	Controls (*n* = 110)	%
**Marital status**	Married	108	99	88	80.0
** **	Single	2	0.9	22	20.0
**Monthly**	≤ 1,500	64	58.2	52	47.3
**Income in Ethiopian Birr**	1,501–3,000	30	27.3	40	36.4
** **	> 3,000	16	14.5	18	16.4
** **	< 20	0	0	1	0.9
**Age (in Years)**	20–30	70	63.6	82	74.5
** **	> 30	40	36.4	27	24.5
** **	Illiterate	20	18.2	29	26.4
	Read and write	22	20.0	7	6.4
**Educational status**	Primary education	9	8.2	6	5.4
** **	Secondary education	33	30.0	24	21.8
** **	College and above	26	23.6	44	60.0
** **	Homemaker	55	50.0	38	34.4
	Governmental	20	18.2	36	32.8
**Occupation**	Private	35	31.8	36	32.8
** **	Gamo	105	95.5	106	96.4
**Ethnic group**	Wolaita	1	0.9	1	0.9
** **	Gofa	4	3.6	3	2.7

### Physical activity Status of participants

**Figure 1:**
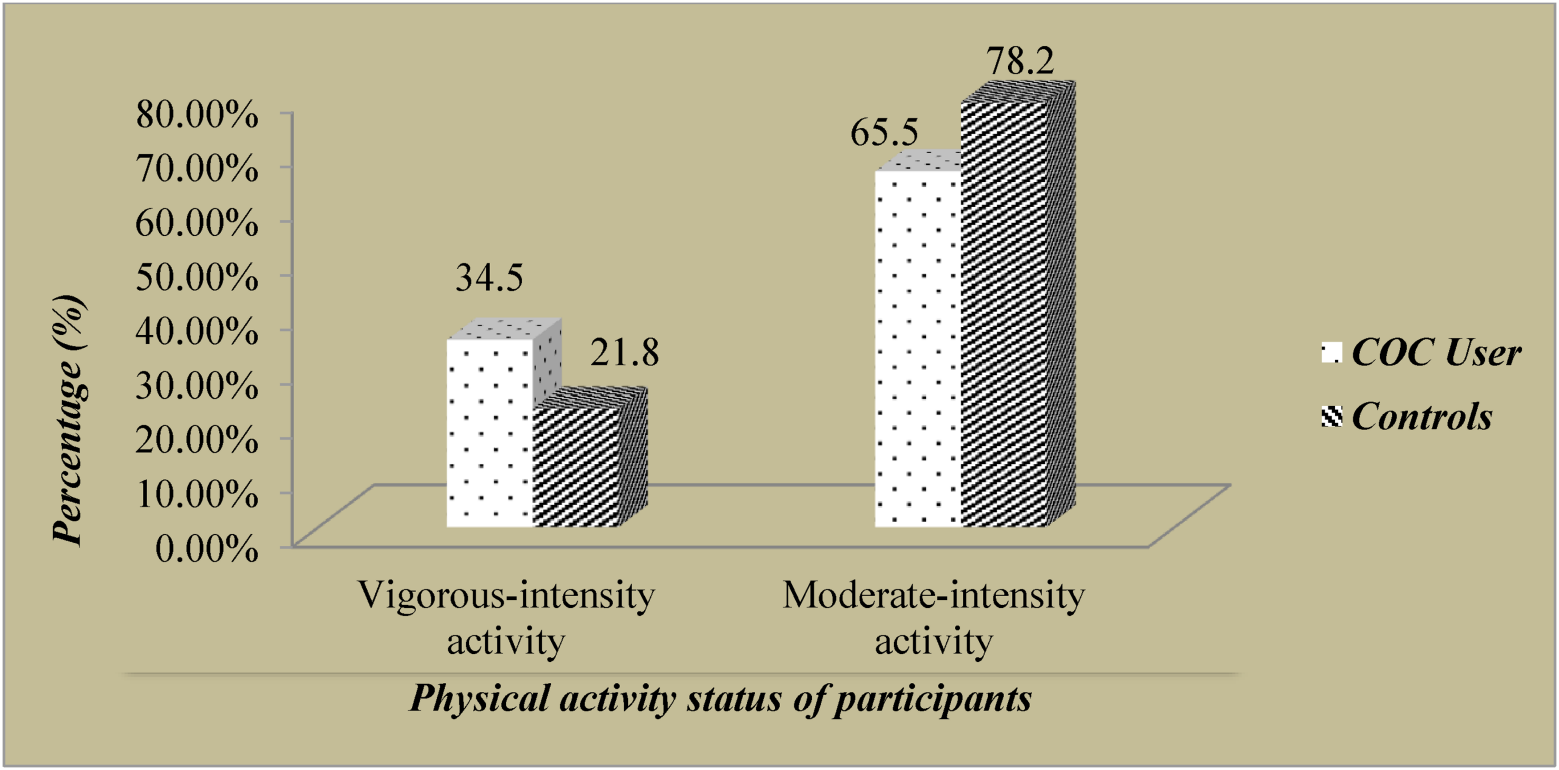
Participants’ Physical Activity status in Chencha town, Southern Ethiopia.

All participants in both groups were physically active. 34.5% of cases and 21.8% of control groups had vigorous-intensity activity in their daily work. Majority of the study participants (65.5% of cases and 78.2% of the controls) had moderate-intensity activities in their daily work (as shown in Figure 1).

### Nutritional Status of participants

**Figure 2:**
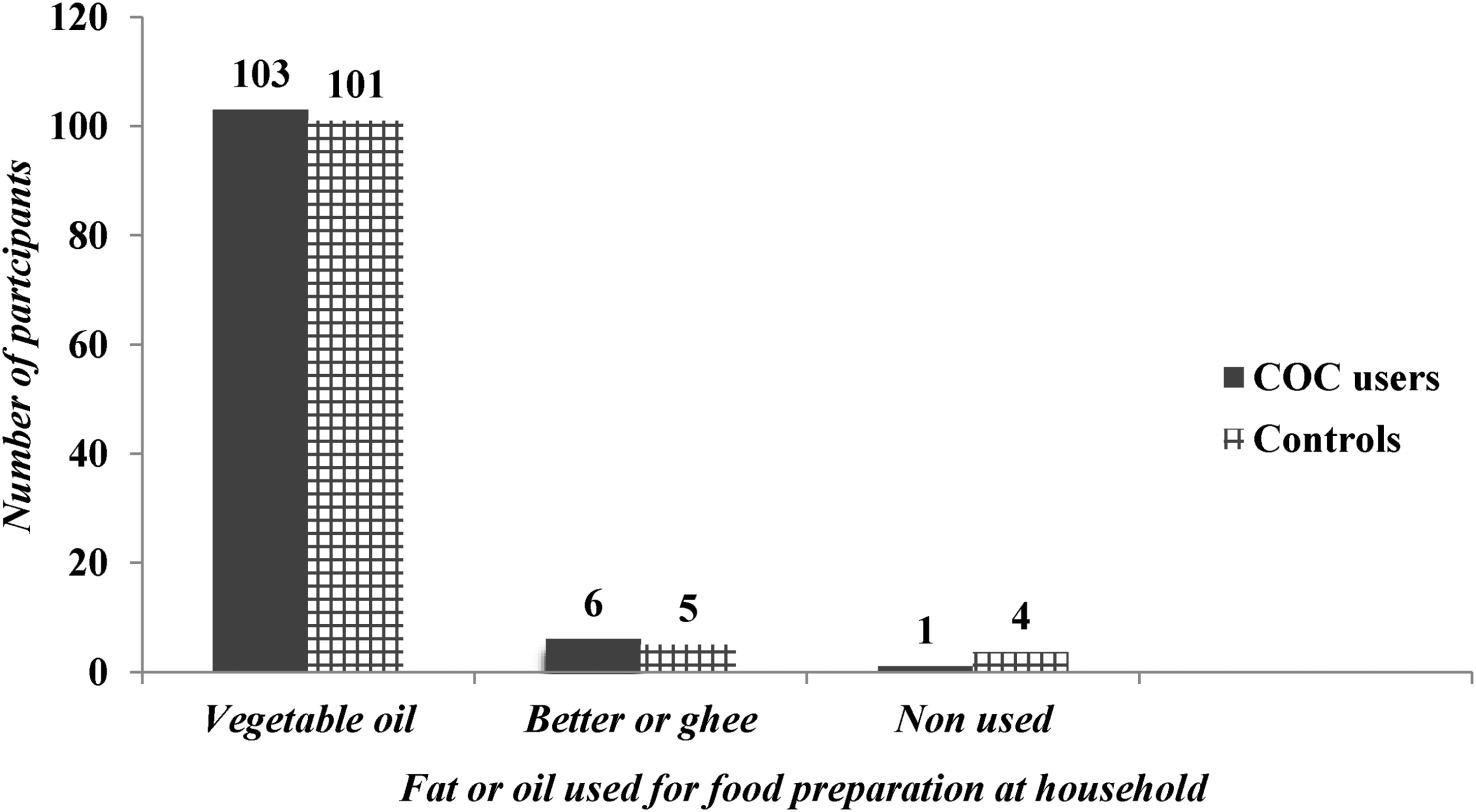
Nutritional Status of OCPs users and controls in chencha town, southern Ethiopia.

Most of the time, 89% and 83% of the cases and controls ate fruit 1–3 days per week, respectively. 79% of cases and 70% of control groups were eating vegetables 4–7 days per week. The majority 93.6% of the cases and 91.8% of controls used vegetable oil for food preparation at home (as shown in Figure 2).

### Use of oral contraceptives pills

In the current study, study participants used OCP on a regular basis for an average of 16 months, ranging from 3 to 36 months. About 16.4% of study participants utilized OCP for more than 25 months; 45.4% of users used it for 13–24 months; and 38.2% of users stayed on the service for 3–12 months.

### Physical and biochemical measurements

The mean FBG level for cases was 88.55 ± 7.89 mg/dl and 86.00 ± 9.85 mg/dl in controls. FBG ranged from 67 to 111 mg/dl among OCP users and 64–109 mg/dl among controls. About 4.5% of OCP users had a FBG value ≥ 110 mg/dl. The MAP among OCP users was higher (88.2 ± 8.5 mmHg) than control group (86.0 ± 6.7 mmHg) with a *P*-value of 0.04 (as shown in Table 2). The mean SBP of cases and controls was (121.8 ± 8.0 and 111.0 ± 8.1 mm Hg) respectively with *P*-value of (*P* = 0.001). The one-way ANOVA analysis showed a significant higher (*P* ≤ 0.05) mean SBP, DBP, and MAP in relation to the duration of OCP utilization among pills users (as shown in Table 3).

**Table 2:** The mean FBG level, MAP, height, weight, and BMI of study participants.

Variables	OCP users (*n* = 110) M ± SD	Control Group (*n* = 110) M ± SD	Mean Difference	Difference	*P*-value
**FBG (mg/dl)**	88.55 ± 7.89	86.00 ± 9.85	2.55 mg/dl	+2.9	0.025*
**SBP (mm Hg)**	121.8 ± 8.0	111.00 ± 8.12	10.8 mm Hg	+9.7	0.001*
**DBP (mm Hg)**	72.8 ± 8.6	74.00 ± 7.06	1.2 mm Hg	−1.6	0.29
**MAP (mm Hg)**	88.2 ± 8.5	86.00 ± 6.74	2.2 mm Hg	+2.5	0.04*
**Height (m)**	1.63 ± 5.8	1.64 ± 5.73	0.01m	−0.6	0.39
**Weight (kg)**	56.5 ± 4.9	55.10 ± 5.40	1.4 kg	+2.5	0.03*
**BMI (kg/m^2^)**	21.3 ± 2.3	20.50 ± 1.82	0.8 kg/m^2^	+3.9	0.003*

BMI, body mass index, FBG, fasting blood glucose, MAP, mean arterial blood pressure, SBP, systolic blood pressure, DBP, diastolic blood pressure. Values are represented as M ± SD, mean ± standard deviation, (+), Increased compared to controls (-) = Decreased compared to controls.

* Statistically significant.

**Table 3:** Mean difference in serum FBG level, SBP, DBP, MAP, weight, and BMI among OCP users with relation to duration of stay on OCP utilization.

Parameters	Duration use (Month)	Frequency (*n* = 110)	Mean	*P*-value
**Change in mean FBG (mg/dl)**	3–12	42	87.3 ± 6.4	0.27
	13–24	50	89.9 ± 5.9	
	≥ 25	18	87.7 ± 13.7	
**Change in mean SBP (mm Hg)**	3–12	42	115.2 ± 5.5	0.001*
	13–24	50	125.6 ± 6.4	
	≥ 25	18	126.7 ± 6.9	
**Change in mean DBP (mm Hg)**	3–12	42	70.0 ± 9.37	0.024*
	13–24	50	74.6 ± 7.6	
	≥ 25	18	74.4 ± 7.8	
**Change in mean MAP (mm Hg)**	3–12	42	83.3 ± 9.45	0.001*
	13–24	50	91.1 ± 6.2	
	≥ 25	18	91.4 ± 6.5	
**Change in mean Weight (kg)**	3–12	42	56.5 ± 4.9	0.46
	13–24	50	56.1 ± 3.9	
	≥ 25	18	57.8 ± 6.9	
**Change in mean BMI (kg/m^2^)**	3–12	42	21.3 ± 2.2	0.03*
	13–24	50	20.9 ± 1.9	
	≥ 25	18	22.6 ± 3.1	

BMI, Body mass index, FBG, fasting blood glucose. Values are represented as M ± SD = mean ± standard deviation.

* Statistically significant.

## Discussions

In this comparative cross-sectional study, FBG level, MAP, mean body weight and BMI were significantly higher among OCP users as compared to their counterparts (as shown in Table 2).

### Fasting blood glucose level

FBG level among OCP users (88.55 ± 7.89 mg/dl) was higher than FBG level among OCP non-users (86.00 ± 9.85 mg/dl) with *p*-value of 0.025. However, it remained in the normal range for all users. Similar study finding was reported by Godsland et al. (21), who observed that OCP increased the incremental areas for plasma glucose by 43% to 61% among OCP users than controls. A study in Finland also found that consumers of OCPs had significantly higher mean serum glucose levels as compared with their baseline values, with an increased risk of developing glucose metabolism abnormalities (22).

The mechanism behind OCP use leads to a higher blood glucose level has not yet been elucidated. The possible mechanism, as demonstrated in rats revealed that the high doses of estradiol decreased the sensitivity of insulin *via* the carbohydrate mechanism (8). Contrary to the present study finding, Berenson and co-workers' (23) reported that changes in the mean FBG level of users of OCPs were not significantly different from controls. This disparity may be due to the variation in the study protocol used in previous and current research. Berenson and co-workers' study was a cohort study involving 703 participants, whereas the current cross-sectional study involved a comparatively small sample size of 220 participants. The discrepancy may also be due to the participants' age difference between the studies. Unlike study conducted by Berenson and co-workers' (23), whose age bracket was between 25 and 33 years, our study participants were aged between 18 and 45 years. Because the women in our sample also include subjects reaching menopause that are predicted to show high FBG levels correlated with age. The disparity in food habits and behaviors can also lead to this discrepancy.

### Use of OCPs and blood pressure

Our current study, showed that the MAP of OCP users (88.2 ± 8.48 mmHg) and systolic blood pressure (121.8 ± 8.0 mmHg) were relatively higher than OCPs non-users (111.00 ± 8.12), with a *p*-value of 0.04 and 0.001 respectively (as shown in Table 2).

Such results from this research were consistent with a study in Pakistan (24) that found significantly higher SBP and DBP among pill users than controls. A related follow-up analysis by Azima and Mousavi (4) found that one year after pill intake, SBP was significantly higher compared to the baseline level. Likewise, the study conducted in Korea (7) found similar results with current research, that prolonged use of OCP is related to higher blood pressure than in non-users. A study on blood pressure in women using OCPs in England (25) found that blood pressure among OCPs users was significantly higher than the controls. A similar study of hypertension among users of OCPs conducted in Texas, United States showed an increased blood pressure in both over-the-counter and clinic users of the pills (26). The reason behind for higher blood pressure among OCPs users might be due to the fact that estrogen containing hormonal contraceptives always change the blood pressure by increasing hepatic production of angiotensinogen, which in turn causes the renin-angiotensin-aldosterone system to elevate the blood pressure (27–29). Furthermore, hypertension has been linked to OCP, especially when used over a prolonged period of time (30).

### OCPs use and body weight and BMI

In our current study, significantly higher mean values of body weight (56.5 ± 4.9) and BMI (21.3 ± 2.3) were recorded among OCP users as compared with non-user controls (55.10 ± 5.40 and 20.50 ± 1.82), with a *p*-value of 0.03 and 0.003 respectively, as shown in Table 2. This is in line with a study conducted in Thailand (31) on the effect of the use of OCPs on BMI and blood pressure, according to which, the use of pills containing estrogen and progestin tends to increase BMI and body weight. Another study of the effect of OCPs on lipid profile, blood pressure, and BMI in Pakistan (24) also showed similar results to this report. Contraceptives induced weight gain could be attributed to fluid retention secondary to the activation of the mineralocorticoid and/or renin-angiotensin-aldosterone system and/or an increase in subcutaneous fat secondary to an increase in appetite and food intake caused by hormones (32) On the other hand, study in Minnesota found that pill users have a higher body weight than their controls, but not statistically significant (33). The disparity found between the studies could be attributed to the difference in race associated with a genetic weight gain predisposition, where black women had a higher mean weight gain compared to white (34).

### Limitations of the study

The potential confounders including total daily calorie intake, physical activity, and intake of cholesterol-rich diets were not measured objectively and the outcome was not compared with the outcomes blood glucose, BP, and BMI. The FBG, MAP, and BMI of OCPs users were not compared with their respective pretreatment values because they were not found on the client's card and registration book. This study is cross-sectional rather than cohort in design, with relatively small sample sizes. So, further longitudinal study with a larger sample size is needed to evaluate the changes in glucose metabolism, blood pressure, and BMI that are associated with use of OCP.

## Conclusions

OCP use for in average more than two years was associated with higher fasting glucose, MAP, body weight and BMI compare to controls. The higher MAP, body weight and BMI among OCP users seemed to be dependent on the duration of OCP use.

## Data Availability

The original contributions presented in the study are included in the article. Further inquiries can be directed to the corresponding author/s.
